# Evaluation of acute and sub-acute toxicity of *Pinus eldarica* bark extract in Wistar rats

**Published:** 2016

**Authors:** Akram Ghadirkhomi, Leila Safaeian, Behzad Zolfaghari, Mohammad Reza Agha Ghazvini, Parisa Rezaei

**Affiliations:** 1 *Department of Pharmacology, Islamic Azad University, Shahreza Branch, Shahreza, Iran*; 2 *Department of Pharmacology and Toxicology, Isfahan Pharmaceutical Sciences Research Center, School of Pharmacy and Pharmaceutical Sciences, Isfahan University of Medical Sciences, Isfahan, Iran*; 3 *Department of Pharmacognosy, School of Pharmacy and Pharmaceutical Sciences, Isfahan University of Medical Sciences, Isfahan, Iran*; 4 *Isfahan Center of Public Health Training and Research, Institute of Public Health Research, Tehran University of Medical Science, Iran*; 5 *Department of Pathology, Seyed-Al-Shohada Hospital, Isfahan, Iran*

**Keywords:** *Pinus eldarica*, *Toxicity*, *Hematology*, *Serum biochemistry*, *Histopathology*

## Abstract

**Objective:**

*Pinus eldarica* (*P. eldarica*) is one of the most common pines in Iran which has various bioactive constituents and different uses in traditional medicine. Since there is no documented evidence for *P. eldarica* safety, the acute and sub-acute oral toxicities of hydroalcoholic extract of* P. eldarica* bark were investigated in male and female Wistar rats in this study.

**Materials and Methods:**

In the acute study, a single dose of extract (2000 mg/kg) was orally administered and animals were monitored for 7 days. In the sub-acute study, repeated doses (125, 250 and 500 mg/kg/day) of the extract were administered for 28 days and biochemical, hematological and histopathological parameters were evaluated.

**Results:**

Our results showed no sign of toxicity and no mortality after single or repeated administration of *P. eldarica.* The median lethal dose (LD_50_) of *P. eldarica *was determined to be higher than 2000 mg/kg. The mean body weight and most of the biochemical and hematological parameters showed normal levels. There were only significant decreases in serum triglyceride levels at the doses of 250 and 500 mg/kg of the extract in male rats (p<0.05 and p<0.01, respectively) and in monocyte counts at the highest dose of the extract in both male and female rats (p<0.05). Mild inflammation was also found in histological examination of kidney and liver tissues at the highest dose of extract.

**Conclusion:**

Oral administration of the hydroalcoholic extract of* P. eldarica* bark may be considered as relatively non-toxic particularly at the doses of 125 and 250 mg/kg.

## Introduction

Herbal medicines are getting considerable interest worldwide. According to the report of the World Health Organization (WHO), herbal medicines are presently used for prevention and treatment of diseases by 80% of people in developing countries. In developed countries, the public interest in herbal prescriptions has also greatly increased (Aschwanden, 2001 ▶). Despite the wide use of medicinal plants, their safety and efficacy have not been fully investigated and more detailed analysis is therefore warranted for evaluation and standardization of herbal formulations (WHO, 2008 ▶). 

Pinaceae or pine family is comprised of several popular conifers with diverse species in 11 genera. *Pinus* as the largest genus in pinaceae family contained numerous long-lived and evergreen trees which broadly spread in many countries including Iran (Farjon, 2005 ▶).

*Pinus eldarica *Medw. (*P. eldarica*) is one of the most common pines in Iran. In traditional medicine, different parts of *P. eldarica* (needles, buds, resin and nuts) are used for the treatment of some diseases including asthma, allergic rashes, dermatitis and wounds (Mamedov and Craker, 2001 ▶; Mamedov et al., 2005 ▶). In pharmacological studies, the blood glucose lowering effect and hypotriglyceridemic and anti-urolithiatic activities have been shown for* P. eldarica* extract (Fallahhuseini et al., 2013 ▶; Huseini et al., 2015 ▶; Hosseinzadeh et al., 2010 ▶). The presence of various biologically active components such as α-pinene, β-pinene, β-caryophyllene, longifolene, α-humulene, δ-3-carene and junipene and high quantities of phenolic compounds including catechin, ferulic acid, caffeic acid, and taxifolin have been recognized in phytochemical analysis of leaves, fruits and bark extracts of *P. eldarica* (Afsharypour and Sanaty, 2005 ▶; Zolfaghari and Iravani, 2012 ▶). 

Since there is no documented evidence for *P. eldarica* safety, the present study aimed to investigate the acute and sub-acute toxicity profile of the hydroalcoholic extract of* P. eldarica *bark in rats.

## Materials and Methods

**Plant material and preparation of extract**

*P. eldarica* barks were collected from Isfahan city (center of Iran) in Isfahan Province in August 2014. Following identification of the plant by a botanist, a voucher specimen (No. 3318) was deposited at the Herbarium of the School of Pharmacy and Pharmaceutical Sciences, Isfahan, Iran. For preparation of hydroalcoholic extract, the barks of *P. eldarica* were powdered to coarse particles and macerated with ethanol (70%) at room temperature for 72 hr and the process was repeated for three times. After filtration (using Whatman filter paper No. 1 (11 µm pore size)), the solvent was removed using a rotary evaporator under low pressure. The obtained viscous residue was freeze-dried and stored at -20 ºC. The yield of the plant extraction was 20 % (w/w).

**Determination of total phenolic content**

The total phenolic content of the hydroalcoholic extract of *P. eldarica* was assessed using Folin-Ciocalteu method which is also called the gallic acid equivalence method. This assay is used for characterization of the plant extract and standardization and normalization of the polyphenols content in the extracts. 

In this method, Folin-Ciocalteu reagent which is a mixture of phosphomolybdate and phosphotungstate was used for the colorimetric evaluation of phenolic and polyphenolic antioxidants only in *in vitro* experiments (Singleton et al., 1999).

Briefly, the plant samples were mixed with sodium bicarbonate (20%). The mixture was then treated with diluted Folin-Ciocalteu reagent. After 2 hr, the absorbance was measured using a spectrophotometer (Bio-Tek, PowerWave XS, USA) at 765 nm. The total phenol content was estimated using a standard curve obtained from various concentrations of gallic acid (50, 100, 150, 250 and 500 mg/l) and expressed in terms of gallic acid as the standard (Yoo et al., 2008 ▶). 

**Experimental animals**

Wistar rats of both sexes (200 ± 20 g, 6 weeks old) were obtained from the animal house of the School of Pharmacy and Pharmaceutical Sciences, Isfahan, Iran and used in acute and sub-acute toxicity studies. The animals were maintained under standard laboratory conditions with 12 hr/ 12 hr light/dark cycles at around 22 °C in polypropylene cages with free access to tap water and standard pellet diet. The experiments were performed according to the international guidelines for laboratory animal use and care. 

**Acute oral toxicity study**

For acute toxicity study, the single test dose of 2000 mg/kg of the hydroalcoholic extract of *P. eldarica* in normal saline was administered to the female and male rats orally using an intra-gastric tube. The rats were fasted overnight prior to extract administration. The control groups received equal volume of normal saline orally. Five animals were used in each control and experimental groups. All rats were observed at 1, 2 and 4 hr after treatment and periodically during the first 24 hr then, daily until 7 days for mortality or any delayed sign of toxicity. Any changes in skin, hair, eyes and mucus membrane, food and water consumption, bodyweight, respiratory rate and behavioral, neurological and autonomic profiles were noted during the test period (OECD, 2002 ▶). Animals were sacrificed under ether anesthesia at the end of the experiment.

**Sub-chronic oral toxicity study**

In sub-acute toxicity study, forty female and male rats (five in each group) received daily oral administration (OECD, 2002 ▶) of the hydroalcoholic extract of *P. eldarica* (125, 250 and 500 mg/kg) or equal volume of normal saline for 28 days (Farah-Amna et al., 2013 ▶). All rats were weighed prior to dosing and every four days during the test period. At the end of the experiment, 12 hr-fasting rats were anesthetized with ether. The blood samples were taken from the heart by direct puncture and collected in tubes containing heparin or EDTA for biochemical and hematological analysis, respectively. The liver and kidneys were removed for histopathological examination.

**Biochemical analysis**

The effects of *P. eldarica* extract on the biochemical parameters were assessed by estimation of the following parameters including plasma alanine aminotrasnferase (ALT), aspartate aminostrasnferase (AST), alkaline phosphatase (ALP), total protein, glucose, urea, uric acid, creatinine, total cholesterol, triglyceride, high density lipoprotein (HDL) and low density lipoprotein (LDL) cholesterol. The parameters were measured by an enzymatic colorimetric method using commercially available kits by a spectrophotometer (Hitachi 902 auto analyzer, Japan).

**Hematological analysis**

The effects of *P. eldarica* extract on the hematological parameters including white blood cells (WBC) and differential leukocyte (neutrophil, lymphocyte, eosinophil and basophil), red blood cells (RBC), hematocrit (Hct), haemoglobin (Hb), platelets count, and mean corpuscular volume (MCV), mean corpuscular hemoglobin (MCH), mean corpuscular hemoglobin concentration (MCHC), mean platelet volume (MPV), and red distribution width (RDW) were estimated using automated hematology analyzer.

**Histopathological examination**

After sacrificing the rats, liver and kidney tissues were collected for the histological studies. The tissues were fixed in 10% buffered formalin, embedded in paraffin, sectioned at approximately 4 μm thickness and stained with hematoxylin and eosin (H&E) for examination by light microscopy. The histological changes in liver and kidney tissues were scored in a blinded fashion as follows: grade 0=normal tissue showing no changes; grade 1= very mild inflammatory changes; grade 2=mild, grade 3=moderate and grade 4=severe inflammatory changes (Arsad et al., 2014 ▶) 

**Statistical analysis**

Values were reported as mean±standard error of mean (SEM). Data analysis was made by one-way analysis of variance (ANOVA) followed by Tukey post-hoc test using SPSS software version 16.0. Pathological scores were analyzed by the nonparametric Kruskal-Wallis test. P < 0.05 was considered to be statistically significant.

## Results

**Total phenolic content of** ***P. eldarica***

The total phenolic content was estimated as 38.1+1.5 % gallic acid equivalents in the extract of *P. eldarica* bark. 

**Acute oral toxicity study of** ***P. eldarica***

Rats receiving the fixed dose of hydroalcoholic extract of *P. eldarica* bark(2000 mg/kg) did not show any clinical signs of toxicity during the observational period of 7 days. There were no abnormal gross findings in the males and female rats. No significant changes in the body weight gains were detected. All animals survived and no mortality was observed until the end of the experiment indicating that the median lethal dose (LD_50_) of *P. eldarica* extract is higher than 2000 mg/kg for both male and female rats.

**Sub-acute toxicity of** ***P. eldarica***

**Figure 1:**
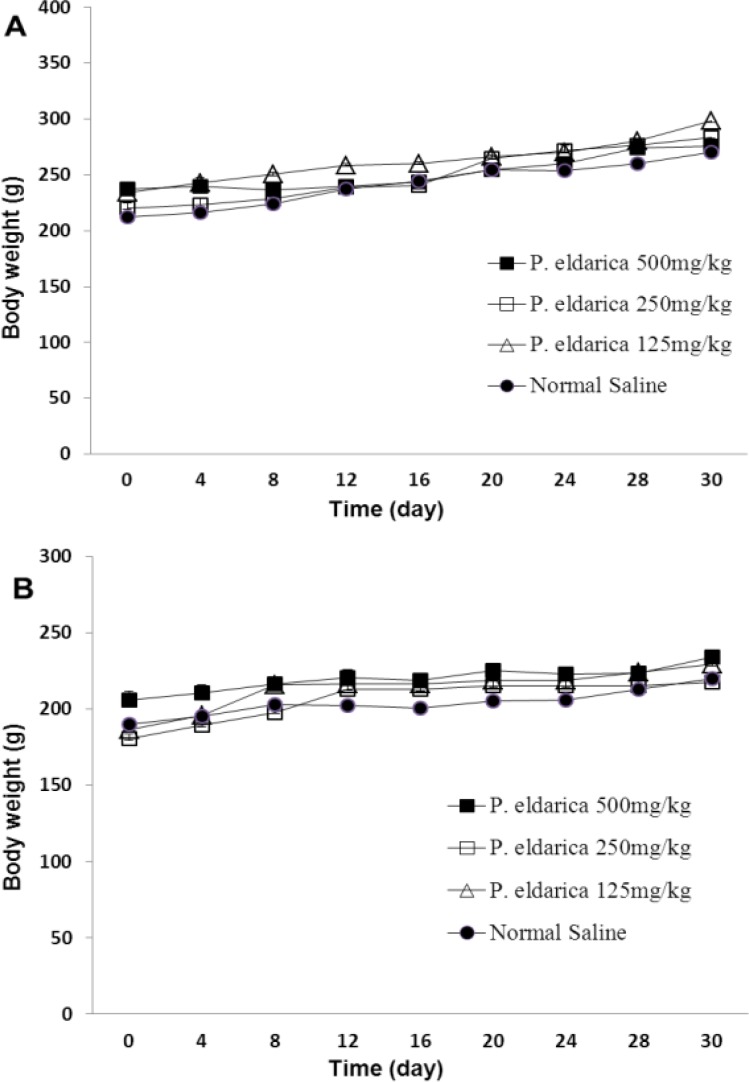
The effect of hydroalcoholic extract of *P. eldarica* (125, 250 and 500 mg/kg) on mean body weight in male (A) and female (B) rats in sub-acute toxicity study. Values are means + SEM for five rats

Here, 28-day administration of *P. eldarica* hydroalcoholic extract (125, 250 and 500 mg/kg) did not cause any mortality in female and male rats. There was no sign of toxicity during the experimental period as compared to the control groups. There was a progressive increase in body weights of control and *P. eldarica* extract-treated rats and no significant difference in the mean body weight was observed between the control and tested animals (Figures 1A and 1B).

In biochemical analysis, a statistically significant decrease in serum triglyceride level was observed at the doses of 250 and 500 mg/kg of *P. eldarica* extract in male rats compared to the control group (p<0.05 and p<0.01, respectively). The effect of sub-acute administration of *P. eldarica* extract on biochemical parameters is presented in Table 1. All other parameters including various lipid markers, blood sugar, total protein, hepatic biomarker enzymes (ALT, AST and ALP), and renal biochemical parameters (urea, uric acid and creatinine) showed normal levels.

**Table 1:** Effect of sub-acute administration of *P. eldarica* extract on biochemical parameters in female and male rats (n = 5

	Male					Female			
	Control		125	250	500	Control	125	250	500
Blood sugar (mg/dl)		157.2 ± 11.29	160 ± 21.28	147.6 ± 0.93	172.6 ± 25.8	198.6 ± 26.10	198.3 ± 23.70	190.4 ± 0.80	191 ± 15.04
Urea (mg/dl)		31.6 ± 4.96	32.8 ± 4.14	33.2 ± 6.47	31.2 ± 3.43	48.8 ± 5.21	44.4 ± 3.37	49.4 ± 4.78	47.5 ± 4.57
Creatinine (mg/dl)		0.74 ± 0.07	0.54 ± 0.07	0.56 ± 0.04	0.54 ± 0.02	0.48 ± 0.04	0.54 ± 0.06	0.72 ± 0.1	0.60 ± 0.05
Uric acid (mg/dl)		0.94 ± 0.05	1.72 ± 0.51	1.36 ± 0.32	1.06 ± 0.22	1.14 ± 0.12	1.28 ± 0.16	1.90 ± 0.36	0.98 ± 0.23
Cholesterol (mg/dl)		90 ± 3.13	97.2 ± 4.48	97 ± 3.24	87.4 ± 1.96	92.8 ± 1.02	98.2 ± 6.5	97.8 ± 6.27	98.1 ± 1.28
Triglycerides (mg/dl)		179.2 ± 19.12	156.6 ± 8.69	120* ± 16.27	103.4** ± 24.7	160.4 ± 18.1	151.6 ± 21.06	159.6 ± 24.2	170.2 ± 21.27
HDL (mg/dl)		38 ± 1.23	41.4 ± 2.32	43.6 ± 3.17	40.4 ± 1.63	44 ± 1.55	37.8 ± 2.35	46.6 ± 4.85	56.4 ± 1,96
LDL (mg/dl)		16.6 ± 3	18.52 ± 1.62	16.78 ± 3.29	16.3 ± 3.29	16.7 ± 3.99	18.1 ± 3.01	16.7 ± 4.45	17.76 ± 3.98
AST (IU/L)		77.8 ±11.33	77.6 ± 24.07	79.4 ± 19.82	71.9 ± 18.81	63.2 ± 21.4	51.6 ± 12.02	52.4 ± 12.1	55.6 ± 9.3
ALT (IU/L)		59.8 ± 7.79	63.2 ± 9.5	61.8 ± 9.3	62.8 ± 9.78	56.1 ± 16.61	48.2 ± 7.54	49.8 ± 19.15	48.6 ± 17.95
ALP (IU/L)		478 ± 44.38	455 ± 55.97	337 ± 54.9	325 ± 39.05	278 ± 56.83	296 ± 69.78	255 ± 44.2	320 ± 62.11
Protein (g/dL)		5.22 ± 0.29	5.68 ± 0.27	5.94 ± 0.23	5.92 ± 0.35	6.0 ± 0.16	5.90 ± 0.17	6.42 ± 0.16	6.44 ± 0.23

* p < 0.05 and

** p < 0.01 show significant differences as compared to the corresponding control.

**Table 2:** Effect of sub-acute administration of *P. eldarica* extract on hematological parameters in female and male rats (n = 5).

	Male				Female			
	Control	125	250	500	Control	125	250	500
WBC (10^3^/μl)	7.52 ± 0.54	7.49 ± 0.56	7.18 ± 1.03	7.23 ± 0.23	5.60 ± 7.10	6.71 ± 0.56	6.70 ± 0.49	5.52 ± 0.81
Neutrophils (%)	12.8 ± 4.88	14 ± 8.3	15.2 ± 6.85	10.4 ± 3.35	9.6 ± 3.76	9.4 ± 4.72	10.2 ± 7.3	9.2 ± 6.25
Lymphocyte (%)	85.4 ± 5.89	76.2 ± 8.26	82.8 ± 7.62	88.4 ± 3.49	88.6 ± 4.61	82.6 ± 5.19	77.4 ± 7.92	76.8 ± 7.18
Eosinophil (%)	0.4 ± 0.25	0.5 ± 0.55	0.8 ± 0.38	0.8 ± 0.36	1 ± 0.55	1.3 ± 0.25	1 ± 0.32	1.2 ± 0.38
Monocyte (%)	1.4 ± 0.78	1.8 ± 0.49	1.2 ± 0.58	0.25* ± 0.6	1.4 ± 0.68	1.6 ± 0.6	2 ± 0.89	0.8* ± 0.86
RBC (10^3^/μl)	7.65 ± 0.12	7.6 ± 0.21	7.8 ± 0.55	7.35 ± 0.21	6.8 ± 0.19	7 ± 0.09	6.7 ± 0.18	7 ± 0.2
Hb (g/dL)	13.54 ± 0.21	12.85 ± 0.65	14.12 ± 0.83	13.3 ± 0.39	11.96 ± 0.64	12.72 ± 0.18	12.52 ± 0.35	12.98 ± 0.24
HCT (%)	38.16 ± 0.69	37 ± 1.77	39.92 ± 2.85	37.35 ± 1.09	34.24 ± 0.88	35.65 ± 0.66	35.57 ± 1.19	35.66 ± 0.89
MCV (Fl)	49.84 ± 0.42	48.17 ± 1.93	50.9 ± 0.3	50.9 ± 0.99	50.1 ± 2.26	50.8 ± 0.74	52.4 ± 0.84	50.56 ± 0.58
MCH (pg)	17.68 ± 0.6	16.72 ± 0.8	18 ± 0.38	18.15 ± 0.34	17.56 ± 1.28	18.19 ± 0.13	18.47 ± 0.28	18.52 ± 0.34
MCHC (g/dL)	34.8 ± 6.93	37 ± 6.57	35.18 ± 0.59	35.4 ± 9.88	34.8± 1.17	38.7 ± 9.87	36.4 ± 1.44	36.4 ± 1.2
Platelets (10^3^/μl)	655 ± 44.43	743 ± 60.09	746 ± 37.42	693 ± 54.5	641 ± 75.84	694 ± 12.68	751 ± 49.05	760 ± 73.22
RDW (%)	15 ± 0.16	16.3 ± 1.51	15.8 ± 0.35	15 ± 0.32	16.3 ± 1.51	14.5± 0.22	14.2 ± 0.35	15.9 ± 0.42

* p < 0.05 shows significant differences as compared to the corresponding control.

**Figure 2.**
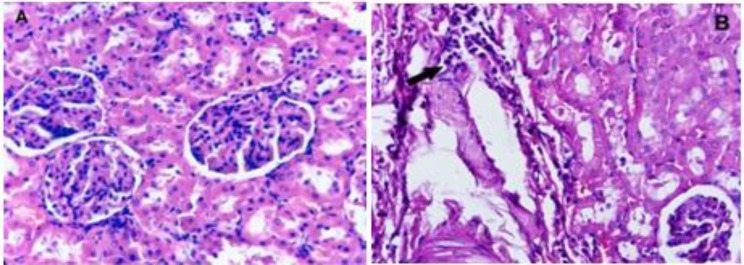
Hematoxylin and eosin stained sections of kidney tissues of control (A) and rats treated with *P. eldarica* extract (500 mg/kg; B) in sub-acute toxicity study (magnification, x400). Mild inflammation with perivascular chronic infiltration is seen in rats treated with high dose of *P. eldarica* for 28 days (Arrow in B

**Figure 3.**
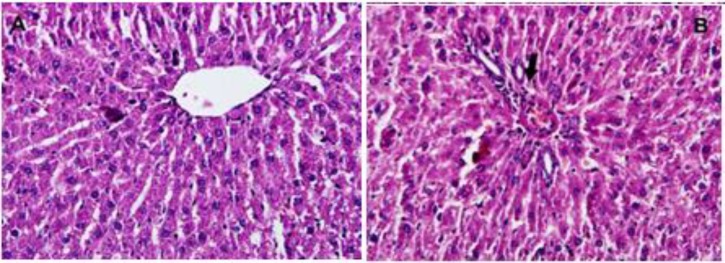
Hematoxylin and eosin staine sections of liver tissues of control (A) and rats treated with *P. eldarica* extract (50=0 mg/kB) in sub-acute toxicity study (magnificatiox400). Very mild inflammation with perivscula infiltration is seen in rats treated with high dose o *P. eldarica* for 28 days (Arrow in B

**Figure 4:**
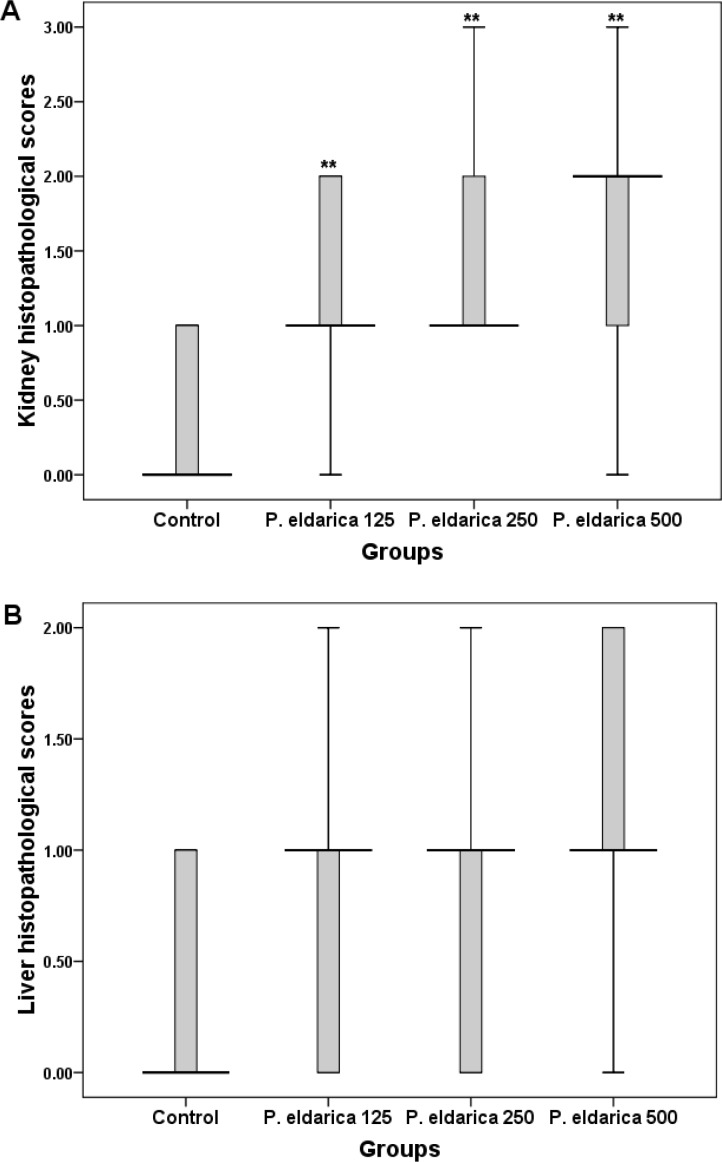
Semiquantitative scoring of kidney (A) and liver (B) histopathologic changes after sub-acute administration of *P. eldarica *extract (125, 250 and 500 mg/kg). Control mice received equal volume of normal saline. Thick lines represent the median of n = 10 (male and female rats), boxes show the interquartile range and bars represent the maximum and minimum sample values. ** p < 0.01 (*Asymptotic significance* level ) as compared to control

Table 2 shows the effects of *P. eldarica* extract on the hematological parameters in sub-acute study. Monocytes were significantly decreased at the dose of 500 mg/kg of *P. eldarica* extract in both male and female rats (p<0.05). All other hematological parameters showed no significant changes and remained within physiological range after the 28-day experimental period. Histopathological examination of kidney tissues following sub-acute administration of *P. eldarica* revealed mild inflammation including acute or chronic infiltration of inflammatory cells especially around large vascular structure and pyelocaliceal system at the dose of 500 mg/kg (Figure 2B). Histological analysis of liver tissues also showed very mild to mild inflammation and slight infiltration of lymphocytes after treatment of rats with 500 mg/kg of* P. eldarica* extract in sub-acute toxicity study (Figure 3B). Figure 4 represents the semiquantitative scoring of histopathological changes in kidney and liver tissues after sub-acute administration of *P. eldarica* extract.

## Discussion

Although herbal medicine as the most common form of alternative medicine are widely used for treatment of various diseases all over the world, their toxicities and side effects are poorly known (Neergheen-Bhujun, 2013 ▶). The presence of many bioactive compounds with mechanisms of action similar to conventional synthetic drugs could predict the potential toxic and adverse effects of phytomedicines (Ogbonnia et al., 2009 ▶). Toxicological cases of aristolochic acids and pyrrolizidine alkaloids and herbal-related nephrotoxicity and hepatotoxicity are well known toxicities of herbal medicine and phytochemicals (Chan et al., 2007 ▶; McLean, 1970 ▶). Allergic responses or anaphylaxis, cardiac toxicity and even carcinogenicity are other examples of dangerous adverse effects following the use of some herbal remedies (Mullins, 1998 ▶; But et al., 1994 ▶; Nortier et al., 2000 ▶). The herbal toxic effects may be related to the intrinsic toxicity or may be due to overdosing, prolonged usage, herb-herb or herb-drug interaction and contaminated formulations (Ko, 1999 ▶). Therefore, precise evaluation of herbal medicines for toxicity and screening of bioactive components should be considered for establishing their safety and efficacy (Ogbonnia et al., 2008 ▶).

This study evaluated the toxicity of oral administration of the hydroalcoholic extract of* P. eldarica* bark in rats. Both sexes of the animals were allocated in each group because toxicological studies have shown small differences in sensitivity between females and males (OECD, 2000 ▶). 

According to the fixed dose method as described in OECD guideline, single doses of 5, 50, 300 and 2000 mg/kg in a stepwise procedure could be used for acute toxicity study. The dose expected to produce some signs of toxicity without producing severe toxic effects or mortality could be selected as a starting dose level (OECD, 2002 ▶). In this study, the limit dose of 2000 mg/kg was used for acute toxicity evaluation because no sign of toxicity has been reported following administration of 500 mg/kg of *P. eldarica* extract in previous pharmacological studies (Bolandghamat et al., 2011 ▶). Our results showed no abnormal gross alteration and no mortality up to the dose of 2000 mg/kg of *P. eldarica*. Single oral administration of substances in the range of 2000-5000 mg/kg bodyweight in rodents is the most common form of acute systemic toxicity testing for defining the range of lethal dose and the effects on important physiologic functions. However, when a limit dose of at least 2000 mg/kg is used and no lethality is recognized, no further testing will be necessary for acute oral toxicity. Due to animal welfare concern, toxicity analysis with dose of 5000 mg/kg is discouraged and should only be used when there is a significant probability for a direct relevance between the results of this test with human health (OECD, 2002 ▶). According to the international standards for classification of hazards, following acute exposure, *P. eldarica* extract could be regarded as relatively low toxic agent since its oral LD_50_ is estimated to be higher than 2000 mg/kg (GHS, 2011).

In sub-acute toxicity study, the effect of repeated oral doses of *P. eldarica* extract for a period of 28 days was evaluated. The long-term toxicity testing could provide data about persistent or cumulative toxic effects on target organ systems, dose-response relationship and also about the no‐observed‐adverse‐effect level (NOAEL) (OECD, 2008 ▶). Our results showed no sign of toxicity and no mortality after repeated administration of *P. eldarica.*

Most of biochemical parameters including hepatic and renal biomarkers showed normal levels proposing no significant adverse effect for *P. eldarica* extract on hepato-renal functions. However, there were some histopathological changes in kidney and liver tissues of the high-dose groups as mild inflammation. These changes may be attributed to the biotransformation and elimination of active constituents of this plant. More studies are required to obtain the pharmacokinetic data of *P. eldarica* extract. Administration of the extract also caused a significant decrease in serum triglyceride levels at the higher doses in male rats suggesting its potential as a possible therapeutic agent in hypertriglyceridemia. Although Fallahhuseini and his coworkers have reported the inability of *P. eldarica* nut extract to change the fasting blood cholesterol and triglyceride levels in hypercholesterolemic diabetic rats (Fallahhuseini et al., 2013 ▶), Huseini et al. have recently shown the efficacy of *P. eldarica* nut on lowering blood cholesterol level in hypercholesterolemic rabbits (Huseini et al., 2015 ▶). 

There are also evidence for beneficial cardio-vascular and cholesterol lowering effects of other species and other parts of the pine such as *P. pinaster* bark extract (Gulati, 2005 ▶). It has been also reported that the ethyl acetate extract of *P. morrisonicola* needle extract has anti-atherosclerotic activity and protective effects against LDL oxidation (Yen et al., 2008 ▶). Interestingly, the hypotriglyceridemic effect of *P. eldarica* extract was found only in male rats in our results. Some evidence has shown sex difference in triglyceride/fatty acid substrate cycling of adipose tissue in rats which is indirectly controlled by androgens (Hansson et al., 1991 ▶).

In hematological analysis, *P. eldarica* extract similarly had no adverse effect on most of hematological parameters in sub-acute study. There was only significant decrease in monocyte counts at the highest dose of extract indicating some mild effect on the blood parameters.

In conclusion, the toxicity data obtained during this study revealed that the hydroalcoholic extract of* P. eldarica* bark is relatively non-toxic followin oral administration especially at the doses of 125 and 250 mg/kg. Regarding the presence of various phytochemical and polyphenolic compounds in different part of *P. eldarica* and several uses in traditional medicine, further detailed investigations are needed to explore the precise safety profile of this plant. 

## Note

*Please cite this paper as: *

Ghadirkhomi A, Safaeian L, Zolfaghari B, Aghaye-Ghazvini MR, Rezaei P. Evaluation of acute and sub-acute toxicity of *Pinus eldarica* bark extract in Wistar rats. Avicenna J Phytomed, 2016; 6 (5): 558-566.
